# Retraction Note: Sirt3 suppresses calcium oxalate-induced renal tubular epithelial cell injury via modification of FoxO3a-mediated autophagy

**DOI:** 10.1038/s41419-020-2318-2

**Published:** 2020-02-10

**Authors:** Yonghan Peng, Cheng Yang, Xiaolei Shi, Ling Li, Hao Dong, Changcheng Liu, Ziyu Fang, Zeyu Wang, Shaoxiong Ming, Min Liu, Bin Xie, Xiaofeng Gao, Yinghao Sun

**Affiliations:** 1 Department of Urology, Shanghai Changhai Hospital, Second Military Medical University, Shanghai, 200433 China; 2 Department of Urology, Zhongshan Hospital, Fudan University; Shanghai Key Laboratory of Organ Transplantation, Shanghai, 200032 China; 3 Institute for Regenerative Medicine, Shanghai East Hospital, Tongji University, Shanghai, 200092 China; 4 Kennedy Institute of Rheumatology, Nuffield Department of Orthopaedics, Rheumatology and Musculoskeletal Science, University of Oxford, Oxford, OX3 7LD UK

**Keywords:** Autophagy, Cell death, Renal calculi

**Retraction to: Cell Death and Disease**

10.1038/s41419-018-1169-6 published online 15 January 2019.

The authors have retracted this article, after publication, because they found that they could not repeat the Annexin V/PI flow cytometry apoptosis experiments with the renal tubular epithelial cells. This method is not stable for CaOx-induced tubular epithelial cells injury detection in vitro. Therefore, the flow cytometry results are not reliable. This specifically impacts Figs. 2a, 3a, 4b, e, 6g and 8a. All authors agree with this retraction.

